# Semi-Automatic GUI Platform to Characterize Brain Development in Preterm Children Using Ultrasound Images

**DOI:** 10.3390/jimaging9070145

**Published:** 2023-07-18

**Authors:** David Rabanaque, Maria Regalado, Raul Benítez, Sonia Rabanaque, Thais Agut, Nuria Carreras, Christian Mata

**Affiliations:** 1 Barcelona East School of Engineering, Universitat Politècnica de Catalunya, 08019 Barcelona, Spain; david.rabanaque@estudiantat.upc.edu (D.R.); maria.regalado@estudiantat.upc.edu (M.R.); raul.benitez@upc.edu (R.B.); sonia.rabanaque@estudiantat.upc.edu (S.R.); 2 Research Centre for Biomedical Engineering (CREB), Barcelona East School of Engineering, Universitat Politècnica de Catalunya, 08028 Barcelona, Spain; 3 Pediatric Computational Imaging Research Group, Hospital Sant Joan de Déu Barcelona, 08950 Esplugues de Llobregat, Spain; 4 Institut de Recerca Sant Joan de Déu, Hospital Sant Joan de Déu Barcelona, 08950 Esplugues de Llobregat, Spain; thais.agut@sjd.es (T.A.); nuria.carreras@sjd.es (N.C.); 5 Neonatal Department, Hospital Sant Joan de Déu Barcelona, 08950 Esplugues de Llobregat, Spain; 6 Fundación NeNe, 28010 Madrid, Spain

**Keywords:** GUI semi-automatic platform, brain segmentation, cerebral ultrasound, preterm, sulci, docker

## Abstract

The third trimester of pregnancy is the most critical period for human brain development, during which significant changes occur in the morphology of the brain. The development of sulci and gyri allows for a considerable increase in the brain surface. In preterm newborns, these changes occur in an extrauterine environment that may cause a disruption of the normal brain maturation process. We hypothesize that a normalized atlas of brain maturation with cerebral ultrasound images from birth to term equivalent age will help clinicians assess these changes. This work proposes a semi-automatic Graphical User Interface (GUI) platform for segmenting the main cerebral sulci in the clinical setting from ultrasound images. This platform has been obtained from images of a cerebral ultrasound neonatal database images provided by two clinical researchers from the Hospital Sant Joan de Déu in Barcelona, Spain. The primary objective is to provide a user-friendly design platform for clinicians for running and visualizing an atlas of images validated by medical experts. This GUI offers different segmentation approaches and pre-processing tools and is user-friendly and designed for running, visualizing images, and segmenting the principal sulci. The presented results are discussed in detail in this paper, providing an exhaustive analysis of the proposed approach’s effectiveness.

## 1. Introduction

Neurodevelopment is a multifaceted process that encompasses the maturation of brain structures, the acquisition of skills, and the shaping of an individual as a unique human being [1]. The brain, composed of countless nerve cells, plays a vital role in centralizing and processing information from our body to generate appropriate responses. This intricate process of brain development is integral to the overall growth and functioning of an individual, contributing to their cognitive, emotional, and physical development. The reasons for preterm birth are multiple and fetal, maternal, and placental factors interact [2]. Some risk factors for preterm birth include chorioamnionitis (infection), maternal hypertensive disorders, genetic factors, altered nutritional status, multiple gestation, and toxic exposure, among others [3]

**Figure 1:**
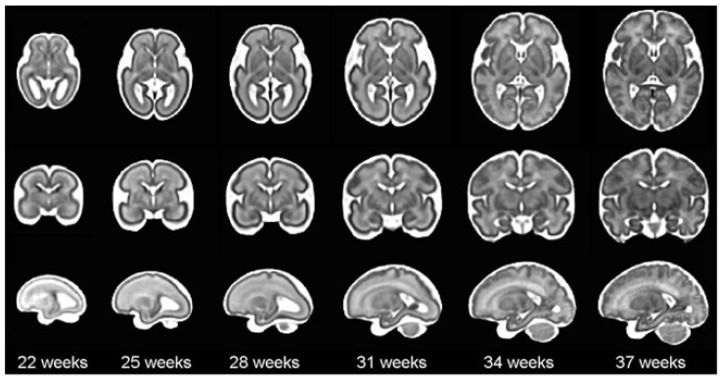
The spatio-temporal fetal brain magnetic resonance atlas (CRL fetal brain atlas) at six representative gestational ages: 22, 25, 28, 31, 34, and 37 weeks. Axial, coronal, and sagittal views of the atlas have been shown for each age point [7].

According to the World Health Organization (WHO), an estimated 13.4 million babies were born too early in 2020; that is more than one in ten babies [4]. Approximately 900,000 children died in 2019 of complications during preterm birth [5]. In this sense, it is worth mentioning that brain maturation is different when comparing a newborn that has completed nine months of gestation with a preterm baby that has grown outside the womb. Preterms are defined as babies born alive before 37 weeks of pregnancy are completed. There are sub-categories of preterm birth based on gestational age: Extremely preterm (less than 28 weeks), Very preterm (28 to 32 weeks), and Moderate to late preterm (32 to 36 weeks). An example of normal brain maturation in fetuses has been described using magnetic resonance imaging (MRI) techniques (Figure 1). To analyze the sulci of the brain during the maturation of the brain, the database is formed by two different planes, coronal or frontal and sagittal [6]. This concept refers to complex changes at specific stages of development whereby the cerebral cortex folds, forming sulci and gyri in order to increase the cerebral surface with a minor increase in volume. The process takes place during the last trimester of gestation, and it follows a specific sequence that allows us to date a specific brain base to its gyration status [7].

Medical imaging modalities commonly used in the pediatric field include X-ray, MRI, ultrasound (US), and nuclear medicine [8,9,10,11]. These modalities provide image sequences that are utilized in the development and validation of new segmentation algorithms. Depending on the specific modality, these algorithms have applications in edge detection, organ localization, region segmentation, and lesion identification [12,13,14,15,16]. These tools, under the supervision of specialized personnel, greatly facilitate the work conducted in hospitals [17,18].

In computer vision, segmentation is the process of dividing an image into different regions based on pixel characteristics, allowing for the identification of objects or boundaries and enabling more efficient analysis of the image. In the medical field, image segmentation plays a crucial role in achieving precise analysis of anatomical data. This involves extracting desired objects from medical images (2D or 3D), which can be accomplished manually, semi-automatically, or fully automatically [19,20,21]. The integration of artificial intelligence techniques provides various solutions. Some researchers have employed methods based on Hough voting and Convolutional Neural Networks (CNN) for the automatic segmentation of objects in MRI images. Studies have also been conducted on brain imaging of premature infants [22], using MRI to compare cortical folding characteristics between healthy fetuses and early preterm infants. This research focuses on a critical developmental period for cortical folding, occurring between 21 and 36 weeks of gestational age (GA). Additionally, spatial and spectral analysis of gyrification has revealed insights into the dynamics of cortical folding waves and deviations associated with prematurity [23]. Finally, a spectral analysis of gyration (SPANGY) method is employed to characterize and track folding progression in preterm infants up to the post-term period.

US is the preferred method for assessing gestational age in newborn brain imaging because it is non-invasive and easily accessible, allowing for repeated and sequential studies directly in the crib or incubator. Healthcare providers should feel comfortable using basic sonographic techniques to determine fetal gestational age, which guides care for both the fetus and the pregnant woman [24]. These techniques are employed to identify and date prenatal or postnatal brain abnormalities, providing sequential visual representations of the infant’s brain. Moreover, automatic segmentation algorithms are used to ultrasound images applying morphological operators [25] in which a method for the automatic measurement of femur length in fetal ultrasound images is presented. More examples are focused on the study of fully automatic segmentation of ultrasound common carotid artery images based on machine learning [26] with the aim of classifying pixels using artificial neural networks to identify the limits of IMT (Intima-Media Thickness). In this sense, the brain imaging was used for a real-time ultrasound segmentation, analysis, and visualization of deep cervical muscle structure [27].

Nevertheless, ultrasound (US) imaging presents challenges in accurately defining the grooves in each plane due to the presence of noise, making image segmentation for groove detection more difficult. As mentioned earlier, various artificial intelligence techniques are employed to achieve automated segmentations [28]. However, a crucial aspect is the utilization of reliable tools that can effectively differentiate and define the distinct grooves for analysis, along with obtaining a well-established ground truth (GT) that has been previously validated by experts. The ability to conduct sequential examinations makes it an excellent tool for studying the in vivo gyrification process in preterm infants.

In order to address this, the Neonatal Brain clinical research group at Sant Joan de Déu Hospital in Barcelona (HSJD) has developed a project specifically focused on brain maturation in preterm infants called BMATURE (Brain Maturation Assessment with Transfontanelar Ultrasound). The primary aim of this research is to develop a graphical user interface (GUI) platform that assists radiologists and medical experts in characterizing brain development in preterm children using ultrasound (US) images. This GUI will involve the study and implementation of various segmentation techniques to create an atlas, enabling the estimation of the baby’s gestational age and facilitating clinical decision-making and proper evaluation of developmental progress.

The specific objectives of this research can be summarized as follows:

- Develop a semi-automatic GUI platform that enables the segmentation of cerebral grooves in ultrasound images.
- Exploring and implementing different segmentation techniques to effectively characterize the development of the brain in preterm children.
- Creating an atlas that incorporates the segmented regions for estimating gestational age and assessing developmental progress.
- Validating the accuracy and reliability of the developed atlas by collaborating with medical experts.
- Contributing to the advancement of artificial intelligence techniques for more effective and efficient segmentation in similar applications.
- Ensure that the platform is easy to use and accessible to medical professionals, even those without extensive image processing expertise.

This paper is organized as follows: in Section 2, we introduce the used dataset describing the implementation and features of the proposed GUI semi-automatic platform. Then, we describe the segmentation algorithms and the available features to make them apply to our database. Finally, we present the methodology, implementation, and the obtained results. In Section 3, we include the results with an experimental case study to demonstrate the advantages of the proposed approach. In Section 4, we discuss the results in the light of the clinical perspective and finally describe the conclusions as well as future works.

## 2. Materials and Methods

In this section, the materials and methods used for the development of the application will be explained. For the first case, the set of ultrasounds images that make up the database of a set of premature babies (Section 2.1) and the software and libraries applied to create the application (Section 2.2) are shown. In the second part, we proceed to explain the methods used to obtain the segmentation of the main furrows and to show them through the app.

### 2.1. Database and Experimental Data

To complete this database, the Neonatal Brain clinical research group at Hospital Sant Joan de Déu Barcelona provided us with brain ultrasound scans from different infants. The ultrasound study protocol involved conducting a scan within the first three days of life, followed by weekly scans until discharge or 40 gestational weeks. All ultrasound images were acquired by an experienced neonatologist (NC) using a microconvex probe (4–9 MHz) Esaote My LabTMAlpha.

**Figure 2:**
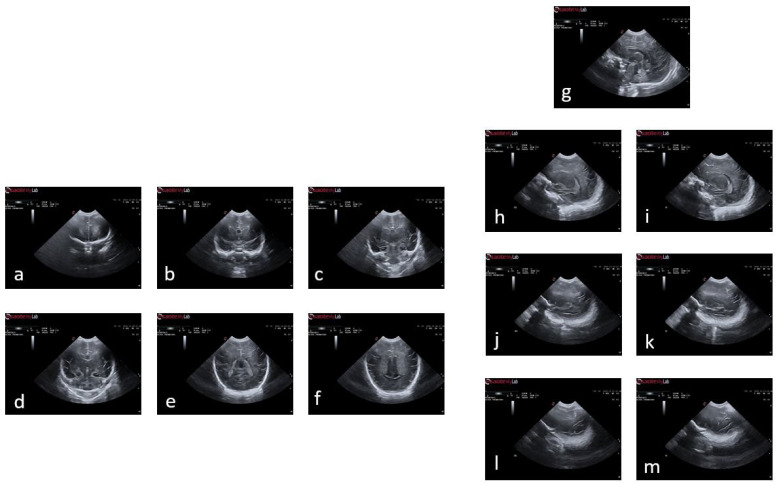
Plans used in the study of a premature baby. For the coronal plane and following the alphabetical order from (**a**) to (**f**), we have the planes of c1, c2, c3, c4, c5, and c6; and following the same order but starting with (**g**) and ending with (**m**), the sagittal planes s1, s2l, s2r, s3l, s3r, s4l, and s4r.

Figure 2 illustrates an example of a premature baby acquisition for both the coronal and sagittal planes. The images of each infant were acquired over several weeks, ranging from 24 to 30 weeks. The images were obtained following anatomical references for different planes:

- Coronal plane: orbital border (c1), sphenoidal ridge (c2), foramina of Monro and third ventricle (c3), fourth ventricle (c4), choroid plexus (c5), and visibility of the parietooccipital sulcus in the inferior tier of the image (c6).
- Sagittal plane: midsagittal (s1), lateral ventricles (s2l, s2r), lateral fissure (s3r), and lateral fissure at the bottom of the image (s4l, s4r).

The timing of the scans varied for each case depending on the clinical condition of the infants. The final repository of images used for the tool included more than 140 subjects, of which 77 were utilized for this study. Infants were excluded if they had brain pathology at birth, intrauterine growth restriction, were born from a multiple pregnancy, or had genetic anomalies or major malformations. All images were anonymized and stored digitally. They were then converted to RGB images in BMP format, measuring 800 pixels in height and 1068 pixels in width. As part of the data processing, these images were pre-processed and converted into RGB images in JPG format, with a size of 550 pixels in height and 868 pixels in width. All images were sorted and assigned an internal code for better identification.

Finally, this project obtained ethical approval from the CEIM Fundació Sant Joan de Déu Ethics Committee in accordance with the requirements of Law 14/2007. The study was assigned the approval code PIC-132-18 and was granted on 03/04/2019.

### 2.2. Graphical User Interface and Software Requirements

This section will explain three different aspects of programming in the project: the coding programs used, the programming languages utilized, and the libraries employed. The libraries will provide pre-existing, complex functions for the programmer to use in the defined code.

#### 2.2.1. GUI Interface Design and Functionalities

To simplify the execution and interaction with the code without the need for direct programming, an interface was necessary for the algorithm. The Dash library was used to accomplish this, as it allows for the creation of interactive platforms that can be accessed through a browser without requiring knowledge of HTML, CSS, or Javascript. Dash offers several advantages, including the ability to work with complementary libraries such as dash-html-components for designing structures, and dash-core-components for basic components such as buttons, plots, and drag-and-drop functionality. Both libraries can be used with Python, allowing for the creation of a platform with its corresponding functions entirely using this programming language.

So, it is important to define the two blocks that make up the platform:

- Layout: describes how the platform is, that is, the appearance it has; and the elements such as graphics, drop-down menus, etc., are defined; and their location, the size of each one of them, the color, etc., in the screen.
- appCallback: is used so that the platform has interactivity with the person who uses it and executes an action or algorithm to obtain a result.

Dash can be divided into two distinct parts: one is focused on defining the aesthetic design of the application, while the other is more concerned with the functional aspects that rely on user interaction.

**Figure 3:**
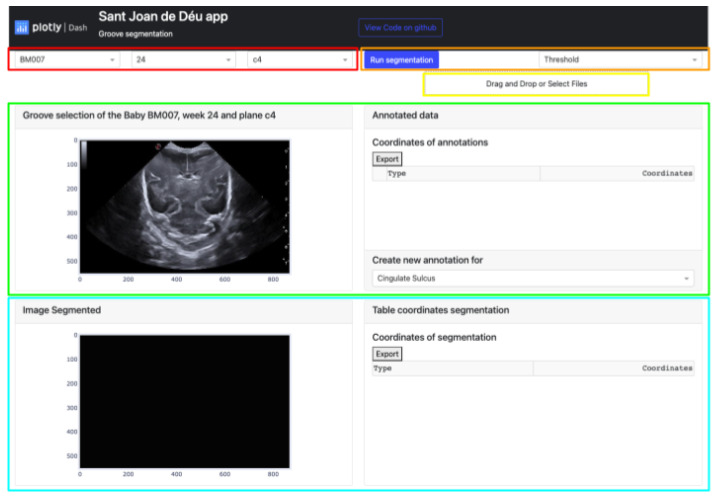
Semiautomatic groove detection platform.

The platform showcases various elements as outlined in Figure 3:

- The buttons that allow the selection of the image from which the manual segmentation is going to be performed. These buttons are adaptive and show the infant identification, weeks, and plane (highlighted in red).
- Located in the top-right corner of the platform are two buttons. The leftmost button displays the type of segmentation applied, which could be either Threshold, Sigmoid + Threshold, or Snake. Clicking the blue button initiates the execution of the selected segmentation algorithm (highlighted in orange).
- The Select Box or Drag and Drop feature enables the import of an exported Excel file and displays it through the upper cards. This allows viewing both the segmentation obtained by certain methods and the corresponding coordinates in the annotation table (highlighted in yellow).
- The top section of the interface contains two cards that handle the segmentation process. The card on the left displays the image selected via the top buttons and enables the user to define the groove area to be segmented. The right card exhibits the coordinates of each manual segmentation executed for each pathway in a table. A button at the bottom enables the selection of the path to be segmented, while the top button exports the table to an Excel file (highlighted in green).
- The lower section of the platform consists of two cards. The first card displays the segmented grooves obtained by the selected method, while the second card displays the numerical coordinates of the defined segmentation. The second card also features an export button that enables users to save the data in an Excel file (highlighted in blue).

**Figure 4:**
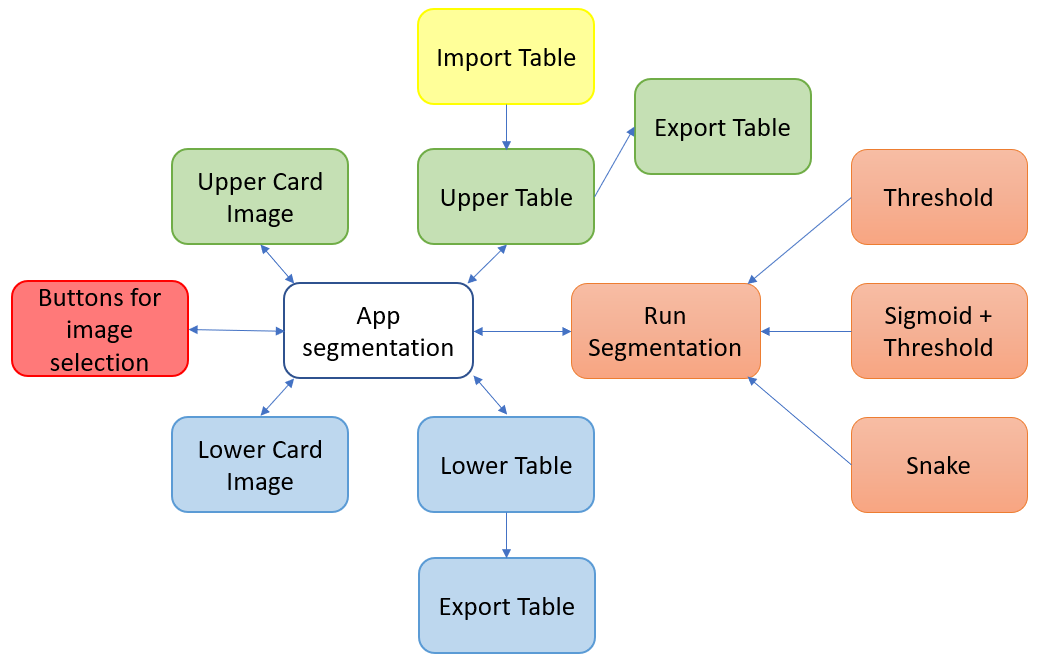
Main software components of the proposed tool.

To facilitate the understanding of these points, a block diagram of the components of the proposed tool is shown in Figure 4.

#### 2.2.2. Software Requirements

This platform is utilized for programming algorithms and functions by writing lines of code. They not only facilitate code execution but also enable result visualization. In the project, two different programs have been employed: Google Colab and Jupyter. Each program has its own advantages, although both are used for the same programming language.

- Cloud: Google Colab platform was utilized to test and evaluate the various methods applied to the images, as their size varied depending on the operations performed, making them too large to be processed locally. As a result, the team opted to work remotely using Google servers.
- Local: Jupyter Lab has been utilized to create and evaluate the platform intended for medical practitioners once its functionalities and design were established. This enables the verification of the performance of both the platform and the algorithm on a local machine.

Python has been the programming language of choice for the project, used for implementing various techniques, functions, neural networks, developing the platform and libraries. These libraries offer a standardized interface for accessing their functionalities, as shown in Table 1.

Each library serves a specific purpose within the project, and their respective fields highlight their area of specialization within Python.

To conclude this section, we will explain the software that will be used by specialists to run the platform. The Docker platform has been used for this purpose. It enables developers to create, run, and scale their platforms swiftly by generating containers. Containers are considered the standard units that bundle the code and all dependencies required for the platform to function reliably and quickly in diverse computing environments. For each container, an image must be created. It is a self-contained, lightweight, executable software package that includes all necessary components for the platform to run, such as its code, runtime, system tools, system libraries, and configurations. Containers are isolated from their environment software, ensuring uniformity in performance despite differences between development and staging. In other words, containers allow virtualization of the operating system or environment rather than the hardware of the machine, making platforms portable and efficient when moved from one device to another.

#### 2.2.3. Methodology Structure and Implementation

##### Step 1: Contrast Enhancement

In all the utilized methods, a cropping operation is performed on the designated groove area, followed by a pre-processing step for the segmented region. This pre-processing involves scaling and adjusting the maximum and minimum pixel values of the obtained images. The largest pixel value is normalized to 1, while the smallest pixel value is normalized to 0. For the remaining pixels, a scaling function is applied, where each pixel in the image is subtracted by the minimum value and divided by the difference between the maximum and minimum values within the clipped image.

(1) $$image=\left(img_{crop}-np.min\left(img_{crop}\right)\right)/\left(np.max\left(img_{crop}\right)-np.min\left(img_{crop}\right)\right),$$

**Figure 5:**
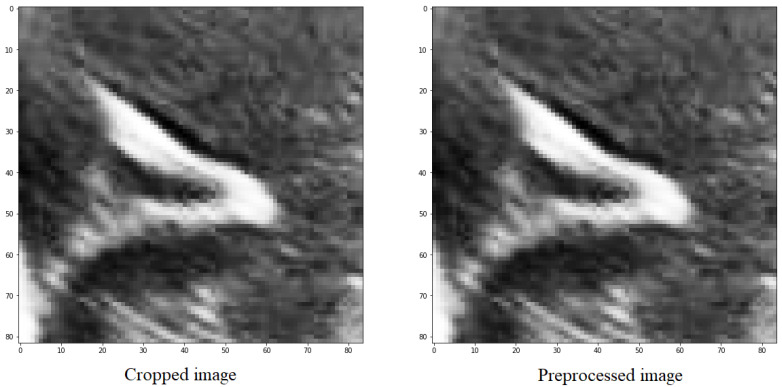
Preprocessing of an image with the objective of scaling the values between the maximum and minimum values of the image.

The purpose of doing this is to distinguish between the structures and the background, as well as the noise present in the image, which is generally darker than the grooves that have values close to 1 (Figure 5).

There exist multiple methods to enhance the contrast between the background and elements in ultrasound images. One such method is Sigmoid Correction, also referred to as Contrast Adjustment.

(2) skimage.exposure.adjust_sigmoid(image,cutoff=0.5,gain=10,inv=False),

Sigmoid Correction transforms the input image pixelwise, according to equation X, after scaling each pixel to the range 0 to 1.

(3) O=1/(1+exp∗(gain∗(cutoff−I))),

The cutoff is the parameter that determines the cutoff of the Sigmoid function (it shifts the characteristic curve in the horizontal direction). The gain, which is the constant multiplying factor in the power of the function. The inv, which is the parameter that, if False, returns the Sigmoid Correction, and if True, returns the negative Sigmoid Correction.

The overall contrast of an image, particularly in the case of ultrasound images, can be improved by applying various treatments that enhance the contrast between the elements and the image background. One such method is the Sigmoid Correction, which is also referred to as Contrast Adjustment.

**Figure 6:**
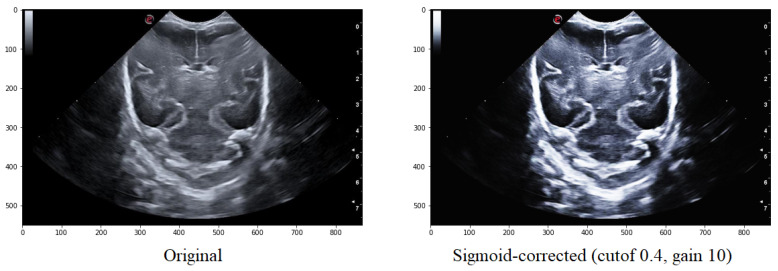
Comparison of the original image with that obtained after applying the Sigmoid function with a cutoff value is 0.5 and the gain value is 10.

Adjusting the cutoff contrast factor and the gain value can control the overall contrast enhancement by regulating the amount of brightening and darkening. At the same time, the default values for cutoff and gain are 0.5 and 10, respectively; they may not always be optimal for the images under consideration. Therefore, several modifications need to be made to determine the best cutoff and gain values that maintain the structure of the grooves while eliminating noise in the surrounding areas. It has been observed that a cutoff value of 0.4 combined with a gain value between 10 helps to eliminate some of the noise around the groove without modifying its structure (Figure 6).

##### Step 2: Binarization

In a grayscale image, which ranges from black to white as the extremes, a higher intensity is inferred when the pixel value approaches 255. This value is derived from the fact that each pixel is represented by an 8-bit binary value, which, when converted to decimal, can only range from 0 to 255.

The threshold method is a technique that enhances and simplifies the identification of structures by leveraging the grayscale and assigning a value of one or zero to each pixel based on the comparison between its value and the predefined threshold. In this case, the brain’s features, such as bones and grooves, have a higher intensity, and their corresponding pixels are assigned a value of one, while brain tissue and fluid, with a darker tone, are assigned a value of zero. Therefore, this method is a viable option and yields satisfactory outcomes.

The platform employs the Local Thresholding method, as ultrasounds are usually noisy, causing intensity to increase in undefined areas. This method allows for the selection of regions (block_size) around each pixel in the input image to determine its value based on the defined method and parameter (param). The “Gaussian” method specifies sigma, while the “generic” method takes a function object that calculates the threshold for the center pixel using the flat matrix of the local neighborhood as a single argument.

**Figure 7:**
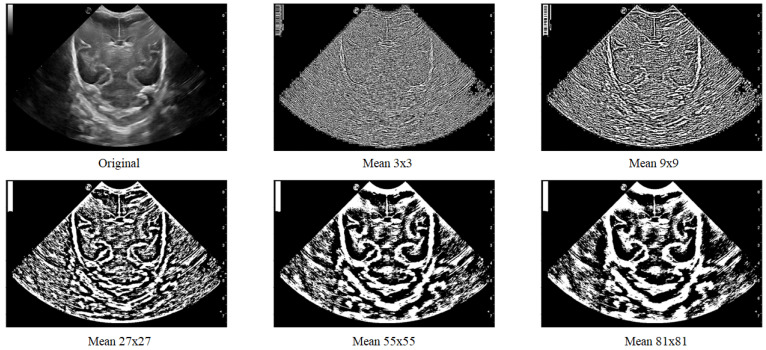
Local filter has been applied to an ultrasound image (Original) with different surface sizes for analysis. As shown in the figure, the resulting images obtained using Mean 3 × 3, 9 × 9, 27 × 27, 55 × 55, and 81 × 81 pixels are presented from left to right as an example.

(4) threshold_local(image,block_size,method,param),

Figure 7 displays various images where local thresholding was performed on different regions. The images show how regions with greater intensity are distinct and separated from other areas where there are no grooves or bones.

##### Step 3: Morphological filtering

Two mathematical morphology operations, erosion, and dilation, can be applied to a binarized image where pixel values are either one or zero. Erosion sets the pixel value at (i, j) to the minimum over all pixels in the neighborhood centered at (i, j), which reduces bright regions and enlarges dark regions. In addition, dilation sets the pixel value at (i, j) to the maximum over all pixels in the neighborhood centered at (i, j), which enlarges bright regions and reduces dark regions.

In the platform, both operations are used in a function called “closing”. This function returns the morphological closure, in grayscale, of an image, defined as a dilation followed by erosion, that can remove small dark spots (known as “pepper”) and connect small shiny cracks. In many cases, this can close the dark spaces between bright features. The function takes the image and the footprint as parameters, with the footprint expressing the neighborhood as a matrix of ones and zeros.

**Figure 8:**
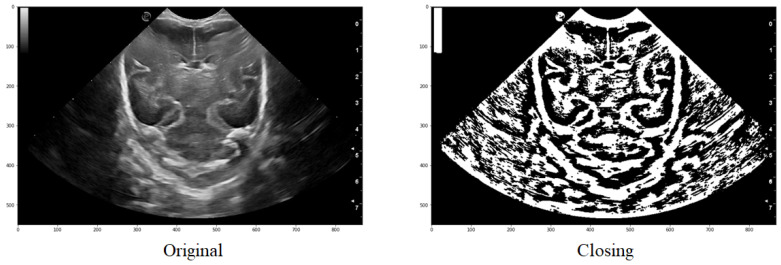
Comparison of the original image with that obtained after applying a threshold and finally the closure function.

(5) closing(image,footprint=None,out=None),

An example of the application can be observed in Figure 8, where it is evident how a group of structures that initially formed part of the same one are joined together to ultimately obtain a structure that is very similar to the one intended to be segmented.

##### Step 4: Object labeling and feature extraction

After binarizing and applying the closing function to the ultrasound image, the identification and characteristics of each structure are obtained using two functions from the Python library skimage.measures: label and regionsprops.

**Figure 9:**
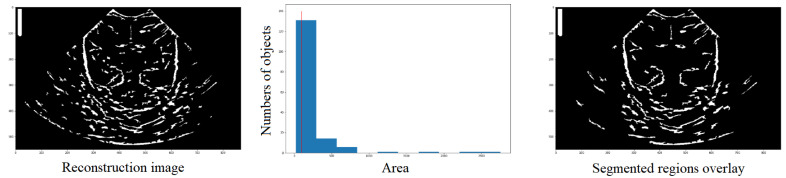
Definition by means of the histogram, of the number of structures that have an area and the elimination of those with an area lower than the reference value (red line), in order to obtain a new image (third column) with those structures that meet the condition.

The label function takes the image to be labeled (label_image) as a parameter and can also take an optional parameter called return_num, which, if set to True, makes the function return the number of regions found. Figure 9 illustrates a visual representation of the segmented regions overlay, where the number of objects (nregions). The output of this function is an image and an integer.

(6) label_image,nregions=label(image,return_num=True),

The label function is useful for recognizing and labeling structures, but it does not provide detailed information about each region, such as its area, centroid, etc. For that purpose, the regionprops function comes into play. It is an algorithm that returns a list with the same number of elements as the number of structures detected in the label_image. Each element in the list contains a dictionary with various characteristics of the corresponding structure, such as area, coordinates, centroid, etc.

(7) $$props=regionprops(label_{image}),$$

##### Step 5: Segmentation

**Figure 10:**
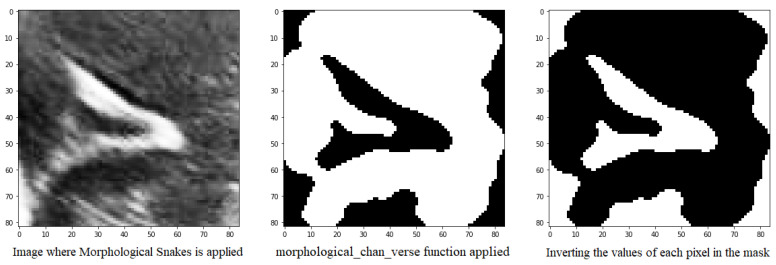
Mask obtained from the morphological_chan_vese function of a groove to be segmented and its corresponding inversion because the number of pixels with value 1 was greater than 50%.

Another method for defining the image furrows is to use the morphsnakes library. It allows segmentation by active contours and is based on Morphological snakes (Figure 10).

Morphological Snakes is a family of methods for the guided evolution of curves and surfaces in images. They have a platform in several areas of computer vision, such as image segmentation and image tracking. The main function used for segmentation is the morphological_chan_vese function that applies the Morphological Active Contours without Edges (MorphACWE) method, i.e., active contours without edges implemented with morphological operators. This technique is commonly utilized for segmenting objects in images and volumes that lack well-defined edges. However, it requires that the interior of the object must be either lighter or darker on average than the surrounding background. It is also robust against noise and can work on images with unclear object contours without any pre-processing.

Upon execution, the function generates a mask of equal dimensions as the input image, which can then be used for segmentation. However, it has been observed that this function may produce errors, as sometimes the path is designated with a value of 0 instead of 1, which can hinder segmentation. To address this issue, it has been proposed to check if the percentage of 1’s in the mask is greater than 50% and invert it if true. This is because the grooves in question are small structures that should occupy less than half of the pixels in the image.

Applying segmentation to an image results in a set of segments that cover the entire image or a set of extracted contours that detect edges. The segments represent regions in which the pixels share similarities in terms of color intensity or other characteristics, and neighboring regions have different characteristics. However, visualizing the entire region is not necessary, and only the contour needs to be highlighted in a specific color, such as red, to identify the structure on the original image easily.

To achieve this, the skimage.segmentation library’s slic function can be used, which uses K-means clustering to segment the objects in the image. The function requires input parameters, such as the image to be segmented, the initial number of labels, an optional mask that calculates only the super pixels where the mask is True (1), and the start label. The function used in the code is as follows:

(8) $$m_{slic}=segmentation.slic\left(img,n_{segments}=2,mask=mask,start_{label}=1\right),$$

**Figure 11:**
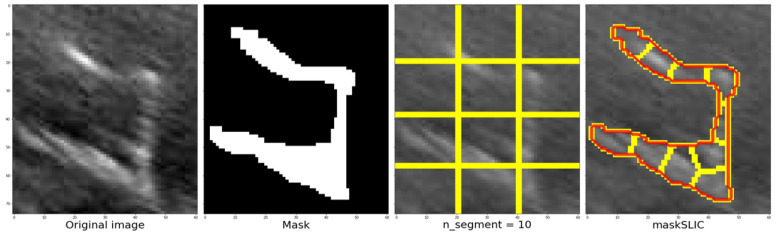
Steps to be followed once the zone has been defined, defining the mask, the number of segments and finally the contour using the maskSLIC function.

The slic function’s process and outcomes are illustrated from left to right, displaying the cropped section of the original image followed by the corresponding mask. Once the image and mask are obtained, the next step is to determine the approximate number of regions using K-means clustering. Figure 11 depicts an example where the image and mask are modified to align with the structure until they correspond. This process enables the grid, highlighted by the yellow line in the third image, to adapt and conform to the structure, ultimately achieving the segmentation of the structure denoted by the red line in the fourth image. In this example, a value of 10 segments was used.

#### 2.2.4. Digital Repository and Distribution

This platform is available in a GitHub repository and distributed with a free open source license on request for access [39]. One of the main aims of this work is to provide a tool for the community to facilitate the use of image-processing tasks. The main idea is to offer this tool to the scientific community in order to improve and incorporate new features and capabilities. For this reason, an open free source and redistribution are available for users. However, the redistribution and use in source and binary forms, with or without modification, are permitted, provided that the following conditions are fulfilled:

1. Redistributions of source code must retain the above copyright notice, this list of conditions, and the following disclaimer [39].
2. Redistributions in binary form must reproduce the above copyright notice, this list of conditions, and the following disclaimer in the documentation and/or other materials provided with the distribution.
3. Neither the name of the copyright holder nor the names of its contributors may be used to endorse or promote products derived from this software without specific prior written permission.

## 3. Results

### 3.1. Experimental Case of Use

We will now explain the structure and design of the platform that has been created using the dash library, as well as how the user interacts with it to ensure the proper use of functions at the desired moment. This way, users can obtain a final result that can be modified according to their needs.

**Figure 12:**
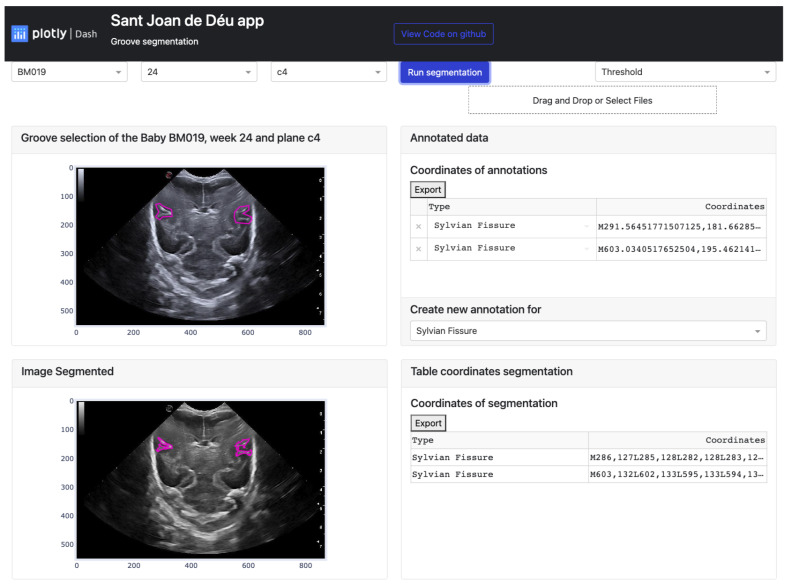
Result of the manual segmentation of each groove defined in the upper cards and carried out by the platform algorithms, in this case Threshold.

Firstly, the platform’s display on the monitor is explained. The interface is user-friendly and straightforward, making it easy to find and display desired information. As seen in Figure 12, the app layout has three rows and two columns. The first row consists of three dropdown buttons on the right-hand side, allowing users to select the baby, week, and database cutoff. On the left-hand side of this row, there are two more buttons. A blue button, when clicked, executes the selected algorithm from the dropdown button below. The algorithm is applied based on the user’s image selection. Below these two buttons, there is another button that allows users to import documents containing groove segmentations.

The second and third rows have a similar structure, with a card displaying a figure plotted in the first column and a second card with a header and a body where the coordinate table is located in the second column. The difference between these two rows is that in the second row, a footer with a dropdown menu to choose the groove type in the original image is also present in the second column.

To summarize, the platform is divided into two main parts. The first part involves selecting the image to be segmented and defining the groove areas, while the second part displays the results of the algorithms once the necessary information is entered. User participation is required for semi-automatic learning in the first part, while the second part allows users to modify the resulting segmentation. To apply the function, users need to click on the “Run Segmentation” button, which has five points defined below:

1. The first step is to select one of the three methods that can be chosen. Each technique performs different steps in processing the image and obtaining the sulcus mask:
  - Threshold: The first step is the rectangular cropping of the image, taking the maximum and minimum values of both axes to perform the scaling. Once this is done, we proceed with the local threshold in order to try to eliminate most of the noise that appears and thus obtain the groove that is being segmented.
  - Sigmoid + Threshold: The process applied when selecting this option is similar to the previous one, but before cutting out the groove area, the image is treated with the Sigmoid Correction function.
  - Snake: In this option, the image, where the furrow has been manually identified, is cropped, and the Morphological Active Contours without Edges (MorphACWE) method is applied. The mask obtained is analyzed, and it is seen if the sum of the pixels that contain the value 1, which corresponds to the groove, is less than 50% of the pixels that the image contains. Otherwise, the mask is inverted, and the pixels that contain the value 1 pass to the value 0 and vice versa.
2. As a second step, we proceed to identify the structures, know the centroid, and the area of each one of them to maintain the structures whose centroid is within the manual segmentation and eliminate the rest. Once the structures that meet the condition have been identified, we proceed to analyze which of these contains a larger surface area in order to eliminate the rest and obtain an image with a black background where only a single structure appears.
3. After obtaining the image from the previous step, the next step is to multiply it with a black-and-white image that defines the structure based on the manual segmentation. The purpose of this image multiplication is to remove the parts of the structure that are outside the manually segmented area, which the user has not marked as part of it.
4. Finally, so that the contour can be shown correctly in the original image, we proceed to adjust the coordinates of the rectangle cropped with the segmentation to where it had originally been cropped in the original image. This is accomplished using the transposition of the image coordinates with the help of the maximum and minimum values of the x and y axes obtained in the first section when cropping.

**Figure 13:**
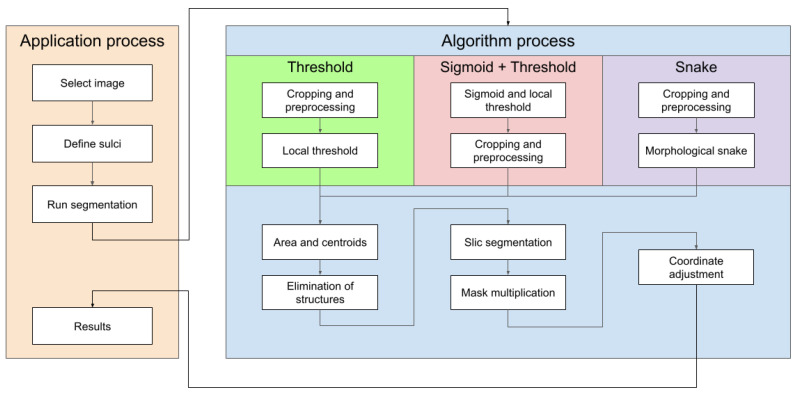
Semiautomatic groove detection platform.

The diagram in Figure 13 shows the steps to follow both in the platform and in the different methods implemented. Here we are able to see the actions to be carried out in any of them and display the results on the screen.

After clicking the button to execute the algorithm, the process is carried out for each manually defined groove, resulting in an image like the one shown in Figure 12. This image displays two identical images of the chosen baby, week, and cut, with the lower image displaying the segmentation obtained by the selected algorithm. The manual segmentation is modified to better fit the shape of the chosen brain groove. The corresponding annotation table, containing the coordinates (in the horizontal and vertical axes) of each vertex that defines each groove, is saved in the left card of its corresponding image.

**Figure 14:**
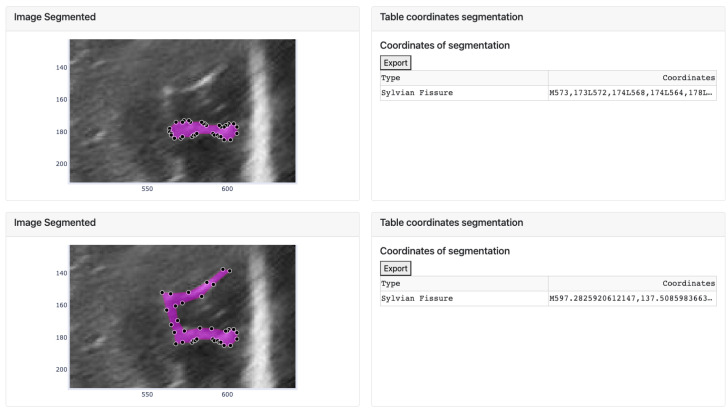
Movement of the vertices of the segment defined by the algorithm and change of the coordinates of the corresponding groove in the table.

However, sometimes the segmentation algorithm fails due to factors such as incorrect separation of the groove from the ultrasound background noise or segmenting it into multiple structures. In such cases, only a part of the groove is segmented (as explained in the third step). To rectify this, the user can modify the segmented image by adjusting the vertices. An example is shown in Figure 14, where the segmentation result is incorrect and requires vertex adjustments. The image shows that some vertices have been moved to new positions to correctly define the groove.

The modifications made to the vectors in the image are reflected in the corresponding table, where the displayed values of the selected coordinates are updated accordingly. Additionally, the data defined in the table can be exported to an Excel document using the “Export” button located at the top of both tables.

**Figure 15:**
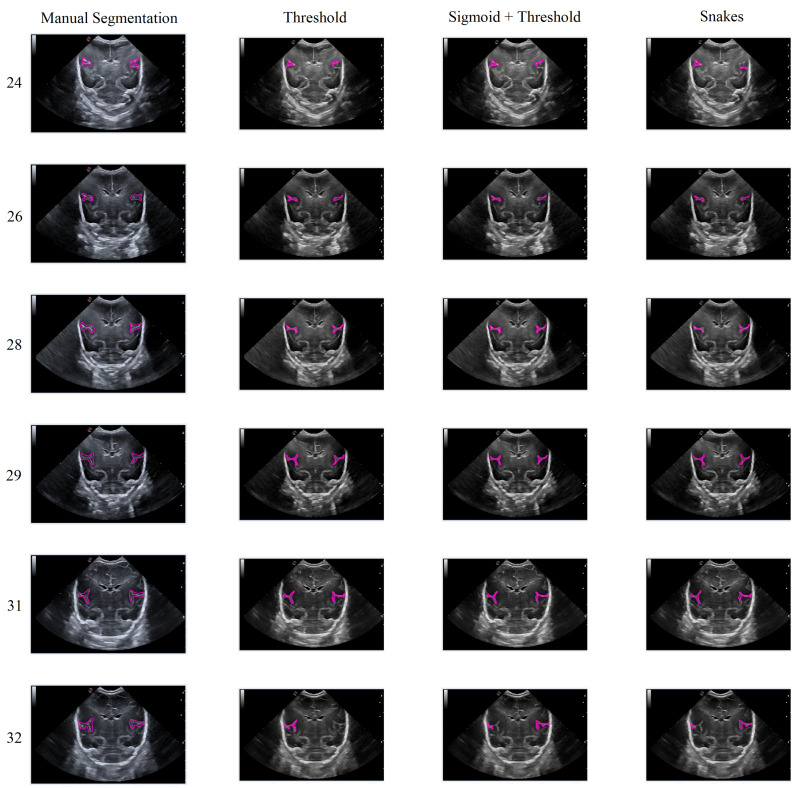
Segmentation examples for the Sylvian sulcus (Manual segmentation), carried out between weeks 24 and 32 of gestation of a mime baby applying the different segmentation methods (Threshold, Sigmoid + Threshold, and Snakes).

In general, the results obtained from the semiautomatic process have been mostly accurate with good precision, except in cases where the noise was too high, making it difficult to identify and define the groove. Nevertheless, the defined algorithm largely fulfills its objective, allowing for modifications to be made to the segmentation results without having to execute the algorithm again. An example of this can be seen in Figure 15, which displays different samples of segmentation on the Sylvian furrow and how it evolves throughout several weeks of gestation in a baby. The figure shows the manual slicer, Threshold, Sigmoid + Threshold, and Snakes algorithms in the columns from left to right. Each row represents the week of gestation when the ultrasound was taken (from top to bottom: 24, 26, 28, 29, 31, and 32).

In general, the algorithms provide good results in cases where the groove is moderately defined, and noise does not affect the structure significantly. However, in areas with high levels of noise, the algorithms tend to struggle to define the groove, resulting in an irregular contour that may not accurately represent the original structure.

### 3.2. Performance Analysis and Evaluation

The proposed segmentation methods have been assessed in terms of their robustness and quality. As shown in Figure 15, segmentation examples for the Sylvian sulcus were generated for the performance analysis. The evaluation of these results involved calculating the Sorensen–Dice Similarity Coefficient (DSC) [40]. The DSC is obtained by computing twice the intersection between the segmentation mask (*S*) and the ground truth mask (GT) and then dividing it by the union of both sets. This coefficient provides a measure of the similarity between the segmentation and the ground truth data. In order to obtain the ground truth, two different medical experts performed the same segmentation process. Subsequently, the segmentation algorithms were compared using the DSC, according to the following equation:

(9) $$\begin{array}{c} DSC=2\times |S\cap GT|/(|S|+|GT|)=\frac{2TP}{2TP+FP+FN} \end{array}$$

DSC involves the consideration of true positive (TP) pixels, which are pixels that have a value of one in both the segmentation mask (*S*) and the ground truth mask (GT). False positives (FP) refer to pixels that have a value of one in the segmentation mask but zero in the ground truth mask, while false negatives (FN) represent pixels that are zero in the segmentation mask but one in the ground truth mask. The DSC coefficient is a numeric scalar or vector with values ranging from 0 to 1. A value of 1 indicates that the segmentation and ground truth masks are identical, reflecting a perfect match between the two.

Furthermore, the time-computing (TC) required for the users to create the segmentations was also evaluated. In order to assess computational efficiency, the time taken to perform the segmentation of an image was measured. This process was carried out using a MacBook Pro 13-inch 2019 model, equipped with an Intel i5 core processor (1.4 GHz, 4 cores) and Intel Iris Plus Graphics 645 (1536MB) with 8GB 2133 MHz LPDDR3 memory. The results of the Dice Similarity Coefficient (DSC) and the TC for different segmentation algorithms evaluated by Expert 1 and Expert 2 are presented in Table 2.

As can be seen, the TC and DSC values are presented for different segmentation algorithms as evaluated by Expert1 and Expert2. The TC is measured in a specific unit (in seconds), and the DSC represents the similarity between the segmentation results and the ground truth. For the Threshold algorithm, Expert1 recorded a computational time of 0.1154 seconds, Expert2 recorded 0.1282 seconds, and the resulting DSC was 0.97054. Similarly, for the Sigmoid + Threshold algorithm, the computational times recorded by Expert1 and Expert2 were 0.1238 and 0.1157 seconds, respectively, with a corresponding DSC of 0.98272. Lastly, the Snake algorithm resulted in computational times of 0.0199 and 0.0184 seconds, as reported by Expert1 and Expert2, respectively. The associated DSC for the Snake algorithm was 0.86256. In this case, the snake segmentation method is dependent on the initial boundary, which explains why the DSC is lower compared to other segmentation techniques. It provides an overview of the computational times and DSC values obtained for each segmentation algorithm, highlighting the performance and agreement between the two experts.

## 4. Discussion

This section will provide an in-depth analysis of the results obtained from a series of segmentation experiments carried out using the three methods implemented in the platform. The grooves will be segmented in the Coronal C4 cut from images of different babies and weeks.

Three different cases will be analyzed. Firstly, the behavior of the algorithms will be observed in different grooves shown in the image. Secondly, an analysis will be carried out on the Sylvian groove to observe how it behaves according to the noise that appears in the image. Lastly, each method’s behavior will be analyzed according to how the manual segmentation has been performed.

**Figure 16:**
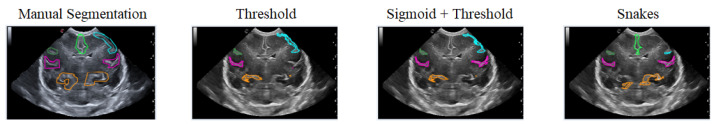
Examples of segmentation of different grooves in an ultrasound scan of a baby at week 29 of gestation and c4 coronal section.

The first case analyzed used an image of a premature infant at week 29 of gestation, in which several furrows have been segmented, as shown in Figure 16. Each color represents a particular groove in which manual segmentation has been applied in the first image or one of the methods applied in the following images, in the order of Threshold, Sigmoid + Threshold, and Snakes.

Figure 16 shows that when applying the Threshold method (second and third images), there is not much variation in obtaining the segmentation. However, applying the Sigmoid preprocessing was able to segment the pink-colored groove on the right side. In contrast, the Snakes method (fourth image) was able to segment an orange-colored groove in the lower right part that had not been segmented with the Threshold method.

In this second scenario, we will analyze the segmentation results of the different methods applied to the Sylvian furrow at four different weeks of gestation (weeks 25, 27, 28, and 30) in various infants. Our aim here is to investigate how the noise present in ultrasound images affects the performance of the segmentation algorithms across different cases.

**Figure 17:**
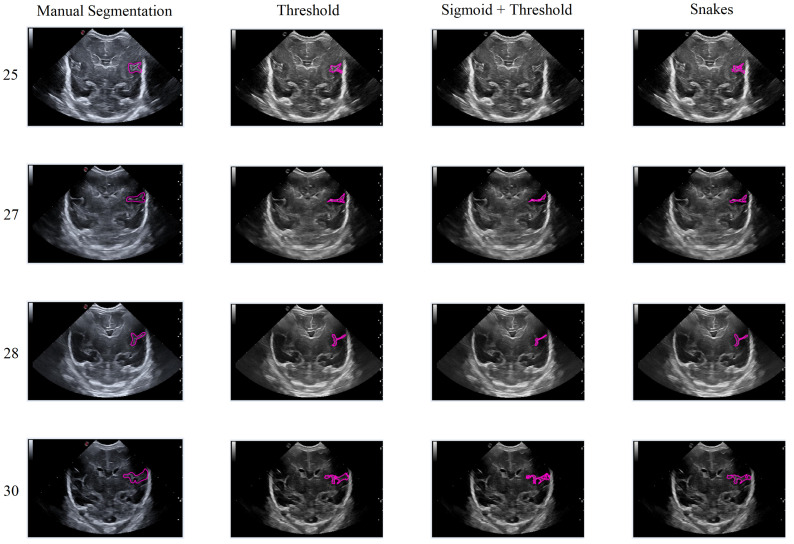
Segmentation of the Sylvian sulcus applying the three defined segmentation methods (Threshold, Sigmoid + Threshold, and Snakes) for different babies and weeks.

In the first row of Figure 17, it was observed that the Sigmoid + Threshold method failed to segment the groove, while the Threshold and Snakes methods were successful, but only if the groove was well defined. In the subsequent rows, which corresponded to weeks 27, 28, and 30 of gestation, a clear difference was observed between the methods that utilized the Threshold (Threshold and Sigmoid + Threshold). The Sigmoid + Threshold method was found to be more sensitive and faithful in segmenting the furrow, but lost precision when defining its shape. On the other hand, the Threshold method lost precision during segmentation but improved the definition of the furrow’s shape.

Another notable observation from the figure is the difference in the way the methods perform segmentation. Specifically, the Snake method produces smoother segmentation results, which appear to be more regular and follow the shape of the groove more accurately. In contrast, when the Threshold method is applied, the segmentation result is more irregular and abrupt, with small peaks visible in the defined shape of the groove. It is evident that in some instances where the groove is more affected by noise, particularly during weeks 27 and 28, the Sigmoid preprocessing can lead to the loss of information about the groove’s shape, resulting in a failure to segment part of it using this method.

**Figure 18:**
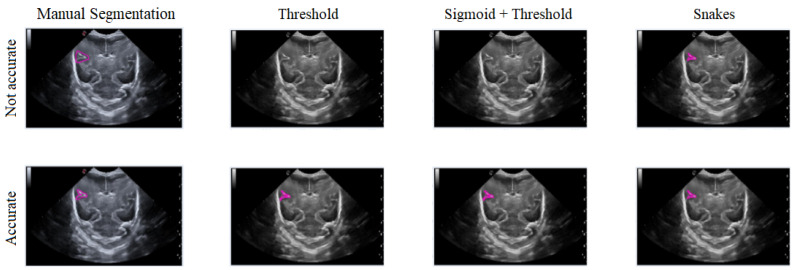
Example of how the segmentation results vary with different methods depending on the accuracy of the manual segmentation, with the first row showing more precise manual segmentation and the second row showing less precise manual segmentation.

In this section, the limitations and considerations were discussed. Each of the three segmentation methods has its own advantages and drawbacks. However, there is a potential loss of precision in defining the shape of the groove, both in its actual form and during the segmentation process. Additionally, ultrasound noise can impact the accuracy of the segmentation, making some methods less suitable. Figure 18 illustrates how imprecise manual segmentation affects the effectiveness of the Threshold and Sigmoid + Threshold methods, while these methods yield satisfactory results when the manual segmentation is more precise. Conversely, the Snake method remains unaffected by the segmentation approach and consistently provides satisfactory results.

When comparing our work to other similar tools, we observe the following distinctions. For example, a review describes the MEDLINE EMBASE database from 2000 to 2018, aiming to account for technical advancements in cranial ultrasound machines and the introduction of MRI imaging in the NICU [41]. However, it does not provide a GUI medical application and is focused on ultrasound imaging. Another example discusses the “Remote Interactive Surgery Platform” (RISP), an augmented reality-based platform designed for surgical telementoring [42]. It utilizes mixed reality head-mounted displays (MR-HMD) and immersive visualization technologies to assist surgeons during operations. Although the RISP offers various features, such as real-time collaboration and three-dimensional annotations, it is primarily focused on surgical applications, and it is out of the scope of our proposal. Finally, the potency and utility of 3D Slicer as an application for prototyping, developing, and evaluating image analysis tools for clinical research applications [43] is well established. Our tool cannot be compared with that in terms of functionality because it is not our intention. We want to make a simple and easy application addressed to the management and segmentation of preterm ultrasound images.

In comparison, our work stands out by offering a user-friendly GUI application that specifically addresses the management and segmentation needs of preterm ultrasound images. It fills a crucial gap in the field and provides a valuable tool for medical experts in their diagnostic tasks. It is important to note that there is no perfect medical tool that can address all the solutions required in healthcare. Each tool has its own limitations and focuses on specific aspects or functionalities. In our case, we have developed a GUI application specifically designed to manage and segment ultrasound images of premature infants. Although our tool fulfills the identified needs and provides a user-friendly interface for medical professionals, it is not intended to replace other existing applications. Instead, it serves as a valuable addition to the medical toolkit, offering a specialized solution for the management and segmentation of these specific images.

## 5. Conclusions

During the third trimester of pregnancy, from week 24 to 40, the human brain undergoes significant morphological changes, including an increase in brain surface area as sulci and gyri develop. However, preterm newborns experience these changes in an extrauterine environment, which can impair brain maturation when assessed at term equivalent age. To address this, a normalized atlas of brain maturation with cerebral ultrasound is proposed in this paper. The atlas enables clinicians to assess these changes weekly, from birth until term equivalent age.

In conclusion, this paper has presented the development of a user-friendly, semi-automatic GUI application specifically designed to meet the needs of Hospital Sant Joan de Déu. The application successfully manages data and creates a validated segmentation atlas for the main cerebral sulci. It is important to highlight that this tool does not aim to replace existing applications but rather fills a specific gap and provides an easily accessible solution for the medical community.

The application offers a variety of segmentation approaches, pre-processing tools, and an intuitive design, enabling seamless execution and visualization of experimental cases. Additionally, the paper provides a thorough discussion of the obtained results, including the analysis and interpretation of segmented images and an evaluation of the proposed approaches’ effectiveness. Comparisons with existing methods and discussions on the clinical implications of the findings may also be included.

Beyond its practical use in managing and segmenting cerebral ultrasound images, this work contributes to the advancement of artificial intelligence in medical imaging. The segmented images serve as valuable resources for training AI models and developing new automatic segmentation tools.

Overall, this work presents a valuable tool for assessing brain maturation in preterm newborns using cerebral ultrasound images. The GUI application and the accompanying results not only enhance our understanding of brain development in this population but also have significant implications for clinical decision-making and patient care. The successful integration of technology and medical expertise in this study opens up new possibilities for improved patient outcomes and future research in the field.

## Figures and Tables

**Table 1:** List of the Python libraries used to implement this medical GUI.

Library	Definition and functions
matplotlib [29]	Library used to generate graphs from data contained in lists or arrays in the Python programming language and its mathematical extension NumPy.
scikit-image [30]	This library is a collection of algorithms for image processing.
OpenCV [31]	Free computer vision library originally developed by Intel.
NumPy [32]	It is a library for the Python programming language that supports creating large multidimensional arrays and vectors, along with a large collection of high-level mathematical functions to operate on them.
PIL [33]	Adds support for opening, manipulating, and saving many different images file formats.
pandas [34]	It is a software library written as a NumPy extension for data manipulation and analysis for the Python programming language. In particular, it offers data structures and operations to manipulate number tables and time series.
keras [35]	Open-Source Neural Networks library written in Python, specially designed to allow experimentation in a more or less short time with Deep Learning networks.
Scikit-learn [36]	Free software machine learning library for the Python programming language. It includes several algorithms for classification, regression, and group analysis, including support vector machines, random forests, Gradient boosting, K-means, and DBSCAN.
scipy [37]	Library that contains modules for optimization, linear algebra, integration, interpolation, special functions, FFT, signal and image processing, resolution of ODEs, and other tasks for science and engineering.
Dash [38]	It is a productive Python framework for creating web analytic platforms that allow a user without programming knowledge to perform tasks already defined through interaction with it through buttons or sliders.

**Table 2:** Computational time analysis (in seconds) and DSC obtained by two expert users for each segmentation technique.

Time-Computing			
Segmentation	Expert 1	Expert 2	DSC
Threshold	0.96677	0.97550	0.99127
Sigmoid + Threshold	0.97124	0.96998	0.99874
Snake	0.99600	0.92772	0.93172
